# It won’t end with COVID: Countering the next phase of American antivaccine activism 2025–29

**DOI:** 10.1371/journal.pgph.0004020

**Published:** 2025-01-08

**Authors:** Peter Hotez

**Affiliations:** Texas Children’s Hospital Center for Vaccine Development, Departments of Pediatrics and Molecular Virology and Microbiology, National School of Tropical Medicine, Baylor College of Medicine, Houston, Texas, United States of America; PLOS: Public Library of Science, UNITED STATES OF AMERICA

## Introduction

Antivaccine sentiments have been expressed throughout American history. However, in this century, the antivaccine movement gained momentum around false claims that vaccines cause autism in the 2000s, followed by “health freedom” protests versus childhood immunization mandates in schools in the 2010s [1]. Starting in 2020 with the introduction of COVID-19 vaccines, health freedom extended to adult immunizations and became a signature feature of political activism on the far-right. This politically charged movement organized and convinced countless Americans to shun COVID-19 immunizations in 2021–22 resulting in an estimated 200,000 deaths from COVID-19 among the unvaccinated U.S. population. Antivaccine activism became a major lethal force in America.

As COVID-19 begins to dissipate and new hospitalizations decline, antivaccine activism has pivoted to childhood immunizations. Like COVID-19, there is a partisan disparity among vaccination coverage, with an August 2024 Gallup Poll finding significantly higher rates of vaccine hesitancy among parents identifying as Republicans [2]. Such parents reported that pediatric vaccines were either unimportant or they presented higher risks due to vaccine side effects than the illnesses the vaccines were designed to prevent. These findings coincided temporally with recent and ominous increases in childhood illnesses, including a five-fold rise in pertussis cases in the U.S. from 2023 to 2024 [3], and the occurrence of 15 measles outbreaks in 2024 (versus four outbreaks in 2023) [4]. Poliovirus was also detected in New York state wastewater following a case of paralytic polio in a 20-year-old unvaccinated man in 2022 [5].

### Delineate the problem

These trends could portend the beginning of regular breakthrough childhood infections due to low immunization coverage. For these reasons, it becomes useful to take stock in the status of childhood immunizations in the U.S. and find new opportunities to advocate for vaccines or counter rising antivaccine activism.

The U.S. Centers for Disease Control and Prevention (CDC) recently published data showing that the state of Idaho at 12.1% has the nation’s highest rate of kindergarten children whose parents request exemptions from school-based vaccine mandates [6]. This number is a potential red flag for highly contagious illnesses such as measles that can spread through schools where vaccination coverage drops below 90–95% [7]. However, just examining rates of state-wide vaccine exemptions does not adequately address the risks of outbreaks or epidemics, since those numbers might mask individual counties where the exemption rate is in fact much higher. Accordingly, my colleagues and I previously wrote to individual state health departments and collected information on 14 states where personal belief vaccine exemptions are permitted by state law for the years 2016–17. Numerous counties were identified where the exemption rate is 5.1 to 30% [8]. They included multiple counties in Idaho, as expected from the recent CDC information, but also many other counties in states ranging from Arizona, to Texas, Wisconsin, and Maine. Several large urban counties also exhibit high vaccination rates.

Having more granular or county-level information would be helpful in assessing pockets or regions of vaccine hesitancy and resistance. Our published data is now more than 6–7 years old. Therefore, the information collected from individual state health departments urgently needs updating and should be expanded to look at all 50 U.S. states. Doing so, might help health policymakers to fully evaluate the areas of the country at greatest risk for breakthrough childhood infections, while also guiding local or state vaccine advocacy initiatives.

### Explaining the risks of illness vs vaccine side effects

**Fig 1:**
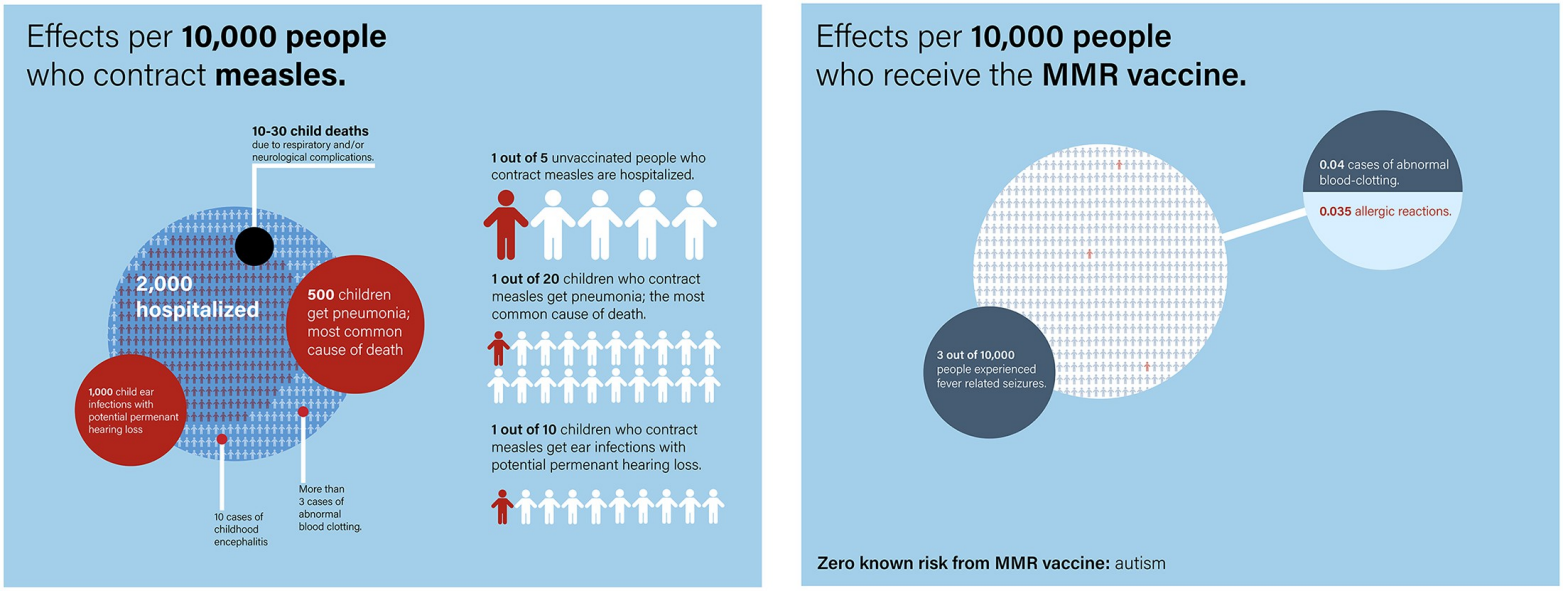
Comparing the risk of 10,000 children acquiring measles (left) versus receiving the measles-mumps-rubella (MMR) vaccine. A new original figure modified from [9]. Artwork by Fahim Akbar.

The next phase in addressing vaccine resistance is to augment existing efforts for vaccine awareness and advocacy. I have found an effective tool for addressing parental concerns—that the risks of vaccine side effects might outweigh the illness—by working in 2020 with the *New York Times* to construct graphics that compare what happens when a child receives a vaccine versus an unvaccinated child acquiring the infection [9]. A new similar graphic is illustrated in Fig 1 for measles and shows what occurs if 10,000 children become infected with measles versus 10,000 children receiving the measles-mumps-rubella (MMR) vaccine.

The published references supporting the data for both the illness and the vaccine are provided in the article, but many of them can be found in *Plotkin’s Vaccine*, initially for the 8^th^ Edition although since then a new edition has been published [10]. Briefly the information and graphic show that if 10,000 children become infected with measles, roughly 2,000 will require hospitalization, 1,000 measles otitis which can lead to hearing loss, and 500 will acquire life-threatening measles pneumonia [9]. In addition, between 10–30 children will die or suffer from encephalitis, although this number is far higher in low-income countries [7, 9]. In contrast, the risks from the MMR vaccines are exceedingly low, with 3 children experiencing febrile seizures and less than even a single child experiencing an allergic reaction or blood clotting. The *New York Times* article contained a similar analysis for influenza versus the influenza vaccine, and for cervical cancer versus the human papillomavirus vaccine [9]. However, I believe such analyses should be undertaken for all childhood immunizations and graphics presented. From my experience this representation has been highly persuasive for parents on the cusp of vaccinating their children.

### Combating disinformation

Beyond detailing the extent of vaccine exemptions and providing accurate information about vaccines and the diseases they prevent we must also show greater resolve at debunking the false statements and disinformation coming from antivaccine activists. Currently, the Health and Human Services (HHS) agencies of the U.S. Government do not routinely identify high-profile pieces of vaccine disinformation and then attempt to correct the science, so as not to inadvertently amplify the misinformed statements. While I understand their rationale, I believe debunking disinformation in real time is essential. For instance, in January 2024 the Florida State Surgeon General called for the halting of mRNA COVID-19 vaccines allegedly because they could integrate into human DNA [11], or cause a “turbo-cancer” [12]. I felt there was a need to publicly refute this and explain the scientific basis of this fallacy. Among my statements made on the cable news channels were to point out that the term “turbo-cancer” is a made-up term, and to explain that mRNA do not typically enter through both the outer cell membrane and nuclear membrane of a cell, which is why electroporation is typically required for DNA vaccine delivery [13]. I also explained how we have proteins of our innate immune, such as interferon-inducible protein 16 (IFI16) that binds to foreign DNA and prevents its integration [14], and even then, the likelihood that this could happen close to a human oncogene is remarkably small. However, relying on a handful of scientists working in universities or academic health centers is not adequate. I propose the establishment of a well vetted publicly accessible website, preferably one created and established by a U.S. Government HHS agency, which can create a continuously updated virtual encyclopedia of vaccine myths and explain in straightforward language why they lack scientific merit.

### Battling at the state level: Do we have the political will?

Still another essential element for maintaining vaccination coverage requires expanding vaccine advocacy and education activities at the state level. In the U.S. most childhood immunization policies are set by state legislatures, with a primary goal to maintain high levels of coverage (90–95%) in schools and prevent breakthrough outbreaks [15]. There are fears that particularly in states where partisan leanings are strong and COVID-19 immunization rates are inadequate, there will be continued declines in childhood immunizations. Therefore, statewide vaccine coalitions and partnerships urgently need help to prevent the passage of onerous legislation that could for example ban pediatric COVID-19 immunizations, require pediatricians to read the full list of excipients in vaccines prior to parental informed consent signatures, stop disease data collection, halt school vaccine mandates, or encourage alternative or unproven immunization schedules. Without question, our system of childhood immunization has been highly successful at eliminating dangerous childhood infections [9]. However, that system now faces an unprecedented political assault that could reverse many of those public health gains. Urgent action is needed to forestall the return of diseases once believed to be consigned to history.
